# Supplementary material for: Comparative multiomics analysis of cell physiological state after culture in a basket bioreactor
Source: Sci Rep. 2022 Nov 23;12:20161. doi: 10.1038/s41598-022-24687-4 (PMC9686226; doi:10.1038/s41598-022-24687-4)

W_023666076.1:8958643:8960308:-@NW_023666076.1:8983503:8983613:-@NW_023666076.1:8983518:898361

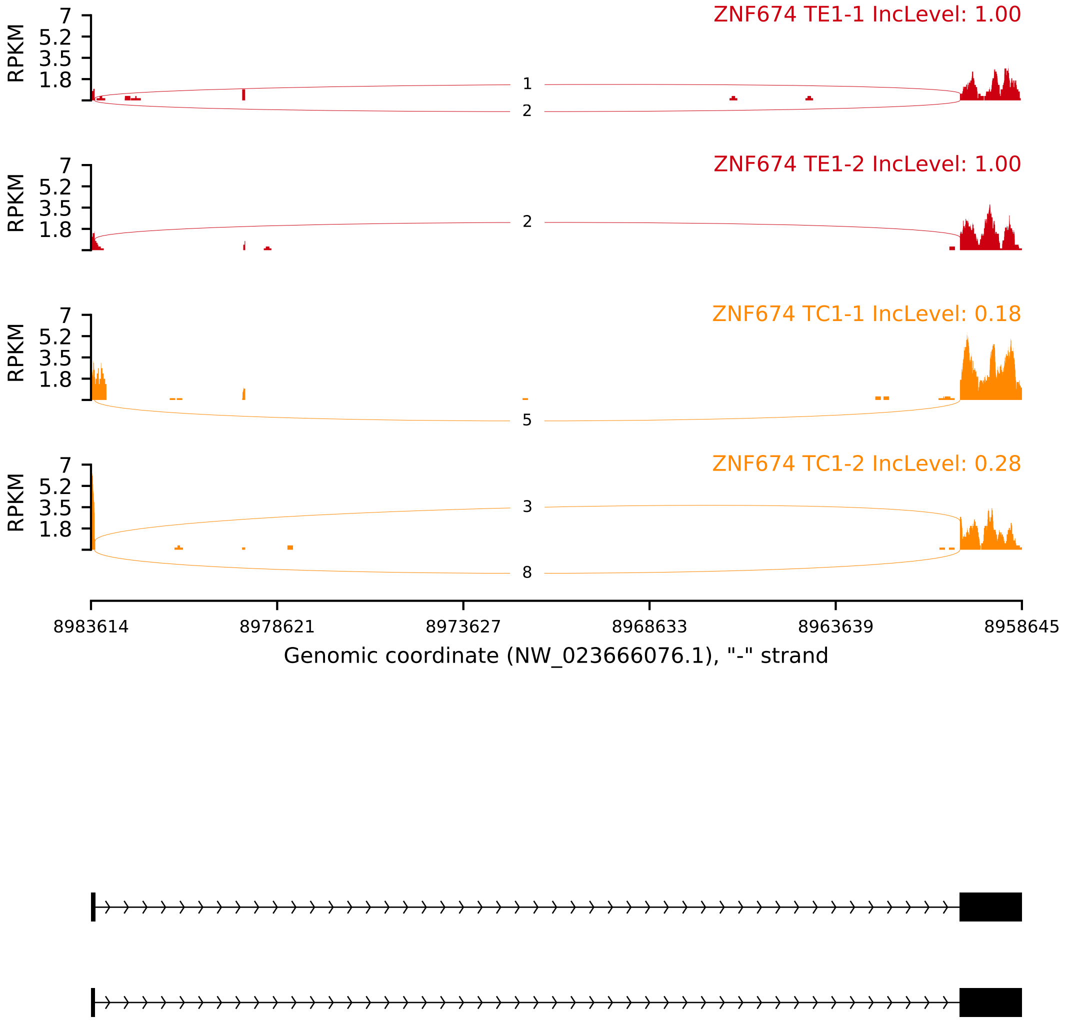

Supplement: Supplementary file 1 — Supplementary Information 1. [file 41598_2022_24687_MOESM1_ESM.zip › raw data/Transcriptomic raw data/7.AS/2.dasplot/TE1vsTC1/A5SS/8_ZNF674_NW_023666076.1_8958643_8960308_-@NW_023666076.1_8983503_8983613_-@NW_023666076.1_8983518_8983613_-.pdf]
